# Recent Advances in Liquid Metal-Based Stretchable and Conductive Composites for Wearable Sensor Applications

**DOI:** 10.3390/bios15070466

**Published:** 2025-07-19

**Authors:** Boo Young Kim, Wan Yusmawati Wan Yusoff, Paolo Matteini, Peter Baumli, Byungil Hwang

**Affiliations:** 1 School of Integrative Engineering, Chung-Ang University, 84 Heukseok-ro, Dongjak-gu, Seoul 06974, Republic of Korea; 2 Department of Physics, Centre for Defence Foundation Studies, Universiti Pertahanan Nasional Malaysia, Kem Sungai Besi, Kuala Lumpur 57000, Malaysia; 3 Istituto di Fisica Applicata “N. Carrara”, Consiglio Nazionale delle Ricerche, CNR-IFAC, Via Madonna del Piano 10C, 50019 Sesto Fiorentino, Italy; 4 Institute of Metallurgy, Metal Forming and Nanotechnology, University of Miskolc, 3515 Miskolc, Hungary

**Keywords:** liquid metal, wearable, composites, sensors, fabrication

## Abstract

Liquid metals (LMs), with their unique combination of high electrical conductivity and mechanical deformability, have emerged as promising materials for stretchable electronics and biointerfaces. However, the practical application of bulk LMs in wearable sensors has been hindered by processing challenges and low stability. To overcome these limitations, liquid metal particles (LMPs) encapsulated by native oxide shells have gained attention as versatile and stable fillers for stretchable and conductive composites. Recent advances have focused on the development of LM-based hybrid composites that combine LMPs with metal, carbon, or polymeric fillers. These systems offer enhanced electrical and mechanical properties and can form conductive networks without the need for additional sintering processes. They also impart composites with multiple functions such as self-healing, electromagnetic interference shielding, and recyclability. Hence, the present review summarizes the fabrication methods and functional properties of LM-based composites, with a particular focus on their applications in wearable sensing. In addition, recent developments in the use of LM composites for physical motion monitoring (e.g., strain and pressure sensing) and electrophysiological signal recording (e.g., EMG and ECG) are presented, and the key challenges and opportunities for next-generation wearable platforms are discussed.

## 1. Introduction

Stretchable electronic devices have gained considerable attention as next-generation form factors due to their superior ability to maintain their electrical and mechanical properties even under tensile deformation. These characteristics enable them to mimic soft and elastic biological interfaces, thus making them promising candidates for bio-integrated electronic systems [1,2,3,4,5,6,7]. The advancement of stretchable bioelectronic devices is expected to reduce the mechanical mismatch between electronics and the human body, thereby enhancing device performance and accelerating the realization of wearable systems for personalized healthcare [8,9].

Extensive efforts have been made to develop stretchable conductors, which are the core components of these systems. Typically, three main strategies have been employed [10]. The first of these involves imparting stretchability to thin metallic films (e.g., gold) by engineering geometrical structures such as serpentine or kirigami patterns; however, this approach offers limited stretchability. The second strategy is to fabricate composite materials by dispersing conductive fillers (e.g., metallic or carbon-based particles) within elastomer matrices; however, this often results in low conductivity [11]. The third and emerging strategy is to use intrinsically stretchable materials, among which liquid metals (LMs) stand out as highly attractive candidates due to their combination of high electrical conductivity and mechanical deformability. Gallium-based LMs provide added benefits, including minimal vapor pressure and favorable biocompatibility for safe integration into wearable and healthcare devices. However, possessing both metallic and liquid properties imposes inherent challenges in shaping and patterning LMs into desired conductor geometries. LMs exhibit low viscosity and exceptionally high surface tension, making it difficult to fabricate thin and uniform films. A simple method to control the shape of LMs is to inject them into microfluidic channels, but alternative container-free approaches focus on tuning interfacial forces. Representative strategies include inducing selective wetting on specific surfaces or incorporating rheological modifiers to adjust the flow and formability of the LM [12].

More recently, the use of liquid metal particles (LMPs) has emerged as a promising solution to overcome the processing limitations of bulk LMs [13]. In particular, LMP-dispersed composites, which integrate the strengths of previously established strategies, represent a significant turning point in the development of stretchable and conductive materials [14]. Hybrid filler composites composed of LMs and other functional fillers have demonstrated outstanding performance and hold broad application potential in wearable electronics [15]. Hence, the present review comprehensively examines recent advances in this field over the past three years, covering the fundamental properties of LMs, the fabrication methods of their composites, and their state-of-the-art applications in wearable sensing devices.

## 2. Fundamentals of LMs and Their Particles

### 2.1. Types and Physical Properties of LMs

**Figure 1:**
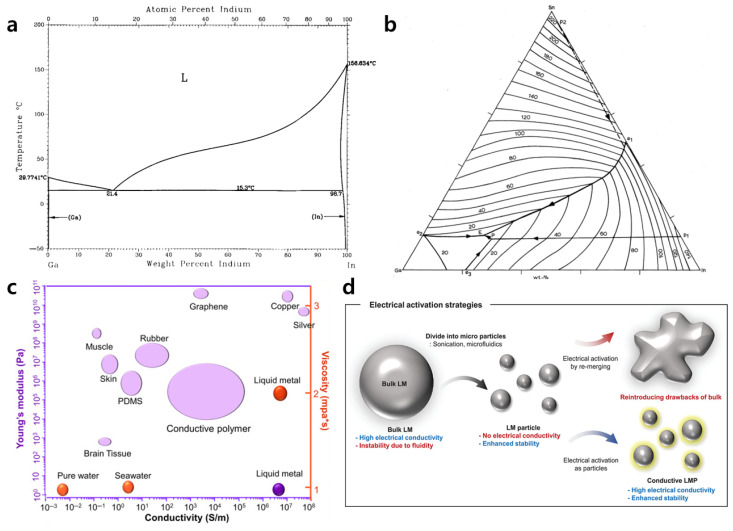
(**a**) The phase diagram of the binary Ga-In system, with a melting point of ~15.3 °C at 75 wt.% Ga and 25 wt.% In [23]. (**b**) The phase diagram of eutectic gallium–indium–tin, with a melting point of ~11° at 66.5 wt.% Ga, 20.5 wt.% In, and 13 wt.% Sn [24]. (**c**) A comparison of the conductivity, viscosity, and Young’s modulus values of common soft materials and a typical LM. Here, the electrical conductivity of the LM is lower than that of copper but significantly higher than that of the conductive polymer [25]. (**d**) A schematic diagram showing the electrical activation of LM particles [26].

The LMs are primarily composed of post-transition metals (e.g., gallium, indium, thallium, tin, lead, aluminum, or bismuth), zinc-group metals (e.g., zinc, cadmium, or mercury), and their alloys. As shown in Figure 1, these materials exhibit a unique combination of metallic conductivity and liquid-phase behavior [16]. Among the elemental metals, only mercury (Hg) and gallium (Ga) are stable in a liquid state at or near room temperature. Unlike Hg, which poses toxicity and vapor pressure concerns, Ga is known for its relatively low toxicity and negligible vapor pressure at ambient conditions, thus making it suitable for bio-integrated applications. Furthermore, Ga exhibits excellent electrical and thermal conductivity. Moreover, alloys such as eutectic Ga–In (EGaIn) and Ga–In–Sn (Galinstan) offer lower melting points than pure Ga, thereby expanding their utility. A notable characteristic of these materials is the rapid formation of a passivating oxide layer (β-Ga_2_O_3_) on their surface, which helps reduce surface tension and plays a crucial role in pattern formation [17]. Since the native oxide skin is extremely thin—typically around 0.7 to 3.0 nm—it is generally not detrimental to the electrical performance. However, it may influence the electrochemical behavior and rheological properties and should, therefore, be considered in practical applications [10]. The physical properties of Ga, EGaIn, and Galinstan are summarized in Table 1.

**Table 1:** The physical properties of liquid Ga, EGaIn, and Galinstan [18,19,20,21,22].

Property	Ga	EGaIn	Galinstan
Melting point (°C)	29.8	15.5	10.9
Boiling point (°C)	2402	2000	>1300
Vapor pressure (Pa)	≈10^−35^ at 29.9 °C	<1.33 × 10^−10^ at 300 °C	<1.33 × 10^−6^ at 500 °C
Viscosity (mPa s)	1.969	1.99	2.09
Density (g cm^−3^)	5.91	6.25	6.44
Surface tension (mN m^−1^)	750	632	718
Electrical conductivity (S cm^−1^)	6.73 × 10^4^	3.4 × 10^4^	3.46 × 10^4^
Thermal conductivity (W m^−1^ K^−1^)	30.5	26.4	25.4

### 2.2. Characteristics of LMPs

Despite the development of various patterning techniques to control the fluidity and high surface tension of bulk LMs, several limitations remain. These include the agglomeration of patterned LM traces during stretching, difficulties in interfacing with microelectronic components, and concerns over leakage [12,26]. One promising approach to overcoming these issues involves breaking bulk LMs into small particles. The resulting LMPs are encapsulated by native oxide shells, which enhance the mechanical durability and environmental stability [13,27]. The oxide shell plays an important role in determining the size and shape of the particles, while also being easily deformable, which is an advantage in many applications.

Bare LMPs are non-conductive due to their discontinuous nature. While this property allows them to function as dielectric materials, they can be reactivated electrically when needed. Under sufficient mechanical pressure—even at room temperature—the oxide shells rupture and neighboring particles coalesce, thus forming conductive pathways [28]. This fusing behavior also imparts self-healing properties to the composites. However, it has been observed that particles smaller than approximately 70 nm cannot undergo sintering at room temperature [29].

An alternative strategy for tailoring the properties of LMPs involves the addition of functional additives to individual particles. These can be selected to enhance the thermal, electrical, magnetic, or biochemical performance according to the specific application [26,30].

An understanding of the physicochemical properties of LMPs—particularly their size-dependent behavior—is essential for practical use. For instance, the melting point tends to decrease as the particle size decreases, thereby enabling liquid-phase retention over a relatively broad temperature range. Moreover, localized surface plasmon resonance (LSPR) phenomena have been reported in the UV range for nanoscale LM particles [31]. Nevertheless, the field of LMPs remains relatively underexplored. For biomedical applications, it is crucial to ensure long-term biocompatibility of LMPs. Long-term implantation or skin-contact scenarios present challenges due to dynamic physiological environments, including mechanical deformation, ion exchange, and enzymatic degradation [32]. To evaluate long-term biocompatibility, international standards such as the ISO 10993-5 [33], which assesses cytotoxicity via direct or extract contact with cell cultures, are widely adopted. Further evaluations may include in vitro and in vivo assessments of immune responses and potential carcinogenicity caused by device materials or leachates [34]. Although previous studies have evaluated the in vivo toxicity of LMPs and reported no essential toxicity, further studies are required to clarify their uptake dynamics and biological pathways quantitatively to enable complete nanotoxicological conclusions [32].

## 3. Fabrication Methods of LM-Based Composites

A useful strategy for utilizing LMPs in a human-compatible manner is to incorporate them into biocompatible elastomers. Stretchable conductive composites, which consist of conductive fillers dispersed within a non-conductive elastic matrix, have emerged as attractive materials due to their exceptional electrical and mechanical properties [35]. Among these, the LM composites represent a new class in which conductive LM droplets serve as the filler phase.

Although the term LM composite has been widely used in the literature to include core–shell structures, LM–polymer composites, and LM–particle composites [25], the present review focuses specifically on the LM–polymer composites. In these, the incorporation of LM droplets into the elastomer matrix makes it possible to tune the material properties while preserving the stretchability. Moreover, the integration of LM droplets with traditional solid fillers can yield synergistic effects, thereby enhancing the overall composite performance [14,15,36].

### 3.1. LMP Formation Methods

The fabrication of LM-based composites typically begins with the dispersion of LM within a polymer matrix. One common method involves mechanically mixing bulk LM with uncured polymer precursors, followed by curing. Alternatively, pre-synthesized LM particles can be blended with elastomer precursors to form a composite material. The latter approach is summarized in Table 2. Two primary strategies are used to generate LMPs, namely, the bottom-up approach and the top-down approach. In the bottom-up approach, nanoparticles are synthesized directly from their molecular precursors, thereby yielding uniform and nanoscale LMPs with controlled size distributions. In the top-down approach, however, the bulk LM is broken down into smaller droplets by the application of physical force, which offers a cost-effective and scalable method for producing large quantities of particles. Among the top-down techniques, sonication is most widely employed due to its simplicity and speed [37].

### 3.2. The Use of an LM as a Single Filler

Initial research efforts on LM-based composites were primarily focused on systems incorporating an LM as the sole conductive filler. When Ga-based LMPs are simply dispersed into an elastomer matrix, the resulting composites generally exhibit high thermal conductivity and electrical insulation [39]. The thermal stability of such composites improves as the particle size of the dispersed LM decreases, while increasing the LM content enhances the material’s dielectric properties [40].

To utilize LM dispersions as electrically conductive materials, post-sintering processes are required. Fassler et al. [41] demonstrated that Galinstan particles embedded in a poly(dimethyl siloxane) (PDMS) matrix could be fused under compressive stress to form stretchable and conductive circuits. Subsequent efforts to apply more precise pressure, such as the use of a pen plotter [42] or embossing [43], have been explored. However, mechanical sintering often causes physical damage to the composite and is not ideal for microscale patterning. To overcome this limitation, various non-mechanical sintering methods have been developed, including the use of a laser [44], temperature [45], acoustic fields [46], and ultrasonication [47]. Despite their effectiveness, these approaches generally require a high LM content (~50%), which increases the risk of leakage during large deformations [48,49].

### 3.3. The Use of an LM as a Hybrid Filler

The incorporation of additional conductive solid fillers enables the formation of intrinsically stretchable and conductive composites. Multiphase composites composed of LMs and traditional conductive fillers such as metals, carbon-based materials, and conductive polymers have demonstrated either enhanced or additional functionalities compared to composites with LMs alone at the same filler loading [15,50]. While extensive research has already been conducted on hybrid fillers in general, the introduction of an LM as a component is relatively recent [48]. The LM not only contributes to the composite’s conductivity but also plays an auxiliary role, i.e., alleviating brittleness by reducing the percolation threshold of the solid filler network [51]. A comprehensive study by Eristoff et al. [15] has summarized the properties, fabrication techniques, and predictive modeling of these “LM + x” composites, where x refers to the solid filler phase. LM and other fillers are typically incorporated into a polymer matrix through simple mixing or emulsification processes. Here, the sequence of material incorporation plays a critical role in inclusion formation and the bulk properties of the LM + x composites. In some cases, LM is pre-synthesized into capsules before being added to the matrix. Additionally, since the density of LM is typically much higher than that of elastomers, it can lead to the non-uniform distribution of LM due to settling during the curing process. The settling phenomena can degrade the electrical conductivity of composites. To suppress settling of LMs, strategies such as pre-mixing the LM with low-density materials or incorporating PDMS beads to induce jamming have been employed [52,53].

However, their analysis lacks any detailed discussion on the critical aspects for real-world industrial adoption, such as process scalability, environmental sustainability, and application-specific integration.

**Figure 2:**
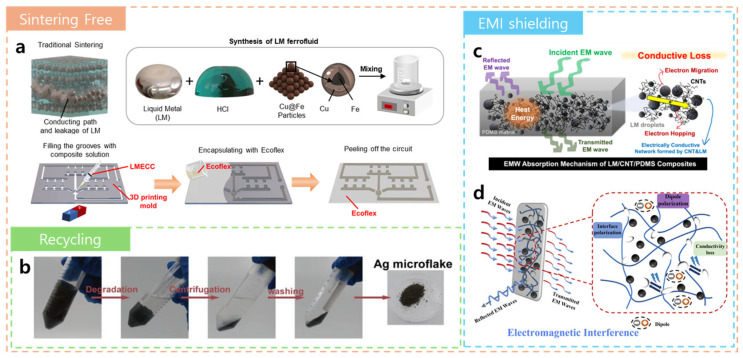
Recent advances in LM hybrid filler composites. (**a**) A schematic diagram showing the preparation of a highly stretchable and conductive LME composite without the need for subsequent sintering (top) and the use of the LM ferrofluid to form a patterned circuit (bottom) [54]. (**b**) A schematic diagram showing the fabrication process of an LM-PHEA-Ag hydrogel and the recycling of Ag flakes [55]. (**c**) A schematic diagram showing the improvement in EMW absorption via a CNT-induced enhancement in the composite’s electrical conductivity [56]. (**d**) A schematic illustration of the EMI shielding mechanism in a PVA/LM/Ni composite film [57].

As most LM-based composites require an additional sintering step, which may affect the long-term stability, alternative methods are being explored. For example, Peng et al. [54] developed an LM ferrofluid by mixing EGaIn with Cu@Fe microparticles (≈10 µm) to achieve conductive networks without the need for a sintering step (Figure 2a). This ferrofluid enabled the fabrication of conductive and stretchable composites (up to ~650% strain) even with low LM loadings. Furthermore, a contactless circuit-patterning method based on magnetic aggregation was proposed. Although the resolution was limited by the mold line width, the selectively magnetized regions formed Janus structures with conductive and non-conductive sides, thus allowing for multilayer circuit architectures.

Environmental considerations, such as the waste treatment and recyclability of LM composites, have become increasingly important. Hence, Zhong et al. [55] developed a biodegradable and reusable hydrogel composed of an LM, poly(2-hydroxyethyl acrylate) (PHEA), and Ag microflakes (Figure 2b). During drying, volumetric shrinkage caused the Ag flakes (≈10 µm) to self-assemble into a percolated network, thereby achieving an electrical conductivity of 1800 S cm^−1^ at an Ag content of 60 wt.% without the need for external sintering. The LM nanoparticles acted as physical crosslinkers, thereby enhancing the mechanical performance of the hydrogel and enabling ultra-high stretchability of up to 1400% strain at 33 wt.% Ag. Remarkably, the hydrogel could be easily decomposed in aqueous NaOH solution, with up to 95% recovery of the silver content.

Stretchable conductive composites can also serve as effective materials for electromagnetic interference (EMI) shielding. EMI shielding mechanisms are typically classified into absorption shielding and reflection shielding [58]. Thus, Kim et al. [56] reported that increasing the carbon nanotube (CNT) content in LM/PDMS composites enhanced the electromagnetic wave absorption due to greater dielectric losses from conductive LM–CNT networks (Figure 2c). This could be attributed to conduction loss mechanisms within the composite [59]. However, excessive CNT loading significantly reduced stretchability, thereby indicating an optimal CNT content of ~1.5 wt.% in order to balance the electrical and mechanical properties.

Unlike CNT–LM hybrids, where absorption effects dominate, pure LM-based composites rely more on reflection mechanisms, especially when the LM droplets have a high aspect ratio [60]. This is because the elongated ellipsoidal LM particles concentrate the electric fields at regions of high curvature, thus leading to stronger polarization loss, while stratified particles increase the multi-reflection and attenuation pathways for electromagnetic waves. Notably, the aspect ratio of the LM has a greater influence on the EMI performance than does the LM content alone. Thus, tailoring the LM morphology and optimizing the CNT loading can provide an effective strategy for EMI enhancement. For instance, Li et al. [57] reported the fabrication of a multifunctional film composed of poly(vinyl alcohol) (PVA), an LM, and Ni particles via a simple physical mixing process. The resulting composite exhibited self-healing ability, EMI shielding, and magnetic responsiveness (Figure 2d). A well-formed conductive network of LM and Ni particles achieved a shielding efficiency of up to 26 dB (over 99% attenuation) in the X-band (8.2–12.4 GHz) frequency range. The dominant shielding mechanism was reflection based, and the EMI performance was well retained after self-healing.

Still, large-scale and reproducible manufacturing of hybrid filler LM composites remains an open challenge requiring further innovation in materials engineering and processing.

## 4. Applications of LM Composites in Wearable Sensors

Wearable devices represent an ideal healthcare platform, offering the capability to be worn directly on the body while enabling continuous and real-time monitoring of health data. Patch-type systems can be adhered directly to the skin to perform a variety of functions, and the intrinsic properties of LMs permit integration into devices without interfering with natural body movements during operation. As a result, LM-based stretchable components such as electrodes [61,62], interconnects [63], and antennas [64] have been actively explored.

To ensure reliable operation under mechanical deformation induced by user motion or environmental changes, the role of the LM composite becomes increasingly crucial [65]. While most published studies have utilized LMs primarily in structural or interconnective roles, relatively few have incorporated LM materials directly into the sensing layer. Nevertheless, stretchable and conductive LM composites are highly promising candidates for application in wearable sensors. Wearable sensors have been expanding across physical, electrical, chemical, and optical sensing modalities [66]. This review excludes chemical sensors, due to challenges related to skin barriers and low reliability, as well as optical sensors, which require additional consideration of optical components. Instead, we focus on two application areas, i.e., physical motion monitoring and electrophysiological signal recording, which not only exhibit higher technological maturity but also effectively leverage the inherent flexibility, conductivity, and self-healing properties of LM composites. Fundamentally, wearable sensors are designed to detect and analyze human motion, which can support a range of applications from personalized fitness tracking and rehabilitation therapy to the early diagnosis of neurodegenerative disorders. Furthermore, if the sensing system can capture physiological signals, it can provide even more detailed and meaningful health-related insights. Several recent studies on wearable sensors and their applications are highlighted in the following sections and summarized in Table 3.

### 4.1. Physical Motion Monitoring

To monitor human motion, posture, and joint bending, wearable sensors must offer both high sensitivity to mechanical stimuli and a wide detection range. Several performance metrics are essential for quantitatively evaluating and comparing the sensor characteristics. For instance, the sensitivity is defined as the slope of the output signal with respect to the input stimulus, with higher values indicating greater responsiveness.

For strain sensors, the sensitivity is typically evaluated according to the gauge factor (GF), which is calculated from Equation (1):

(1) $$GF=(\frac{\Delta R}{R_{0}})/\varepsilon$$

where $\Delta R/R_{0}\;$ is the relative change in electrical resistance, and ε is the applied strain. In the case of pressure sensors exhibiting piezoconductivity, the sensitivity (S) is calculated from Equation (2):

(2) $$S=(\frac{1}{\sigma })(\frac{\partial \sigma }{\partial p})$$

where σ denotes the electrical conductivity, and p is the applied pressure.

Another important factor is hysteresis, which affects both accuracy and repeatability. Lower hysteresis contributes to the viscoelasticity of the polymer composite, thereby indicating better mechanical response under deformation.

**Figure 3:**
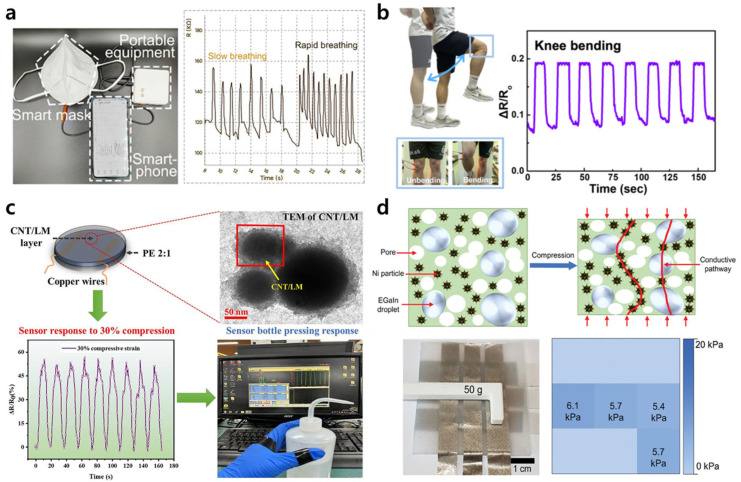
Applications of LM composites in physical motion monitoring. (**a**) An LM/PPy/TPU composite membrane attached inside an N95 mask (left) to monitor the variation in relative resistance during human respiration, with the data (right) being accessible via a smartphone [67]. (**b**) The relative resistance response of an LM/CNT/PDMS composite-based strain sensor to knee movements [56]. (**c**) A PET/CNT/LM/PE composite sensor (upper left), along with a TEM image of the component CNT/LM particles (upper right), a graph showing the electromechanical performance under 30% compressive strain (lower left), and a photographic image showing the real-time measurement of physical movement (bottle pressing) [68]. (**d**) A schematic illustration (top) showing the positive piezoconductive effect of a porous composite due to enhanced conductive pathways under compression, along with a photographic image (bottom left) and corresponding plot (bottom right) showing how the composite-based 3 × 3 sensor array enables the real-time sensing of pressure distribution [69].

Du et al. [67] proposed a strain sensor based on conductive polypyrrole (PPy) bonded to a thermoplastic polyurethane (TPU) fiber pad in which the LM was uniformly dispersed. The resulting LM/PPy/TPU sensor exhibited an exceptionally low detection limit of 0.25% strain, along with a high GF of 4.36 within a strain range of 0–12.5%, making it suitable for the detection of subtle body deformations. As shown in Figure 3a, the sensor was successfully integrated into a medical face mask to demonstrate the real-time monitoring of respiration. Meanwhile, Kim et al. [56] demonstrated a CNT/LM/PDMS composite with both excellent EMI shielding and an outstanding strain sensing performance (Figure 3b). This sensor exhibited a low hysteresis in the 0–80% strain range and a GF of 5.35 at strains of 50 to 100%, which is one of the highest GFs reported for LM-based strain sensors. Moreover, it showed a stable performance in monitoring joint movements at the neck, elbow, knee, and wrist.

In another study, Kouediatouka et al. [68] developed a pressure sensor in which a conductive CNT/LM layer was sandwiched between two layers of 2:1 PDMS/Ecoflex (“PE”) (Figure 3c). Pre-forming the CNT/LM particles improved the CNT dispersion and simultaneously suppressed LM surface oxidation, thereby providing a synergistic effect. The resulting PE/CNT/LM/PE sensor achieved a GF of 57 at 30% deformation, along with a response time of 70 ms and a maximum load capacity of 2451 N. The reconfiguration and fluidity of the CNT/LM ensured strong recoverability even under repetitive compression, thereby validating its potential in pressure sensing applications.

Porous composites provide another strategy for enhancing both flexibility and pressure sensitivity by lowering the elastic modulus [72]. For instance, Stevens et al. [69] optimized the pore size and porosity of an LM composite with a composition of 0.8:6:1:1 1,2-propanediol:nickel:EGaIn:PDMS (by mass) to achieve a high pressure sensitivity (Figure 3d). Compared to conventional materials, this composite showed superior performance in terms of both sensitivity and sensing range, as well as maintaining its signal stability after 1000 compression cycles. A pressure sensor array based on this material successfully detected both the magnitude and spatial distribution of pressure with a resolution below 0.1 kPa and functioned reliably even on surfaces with a curvature radius exceeding 1.6 cm.

### 4.2. Electrophysiological Signal Recording

**Figure 4:**
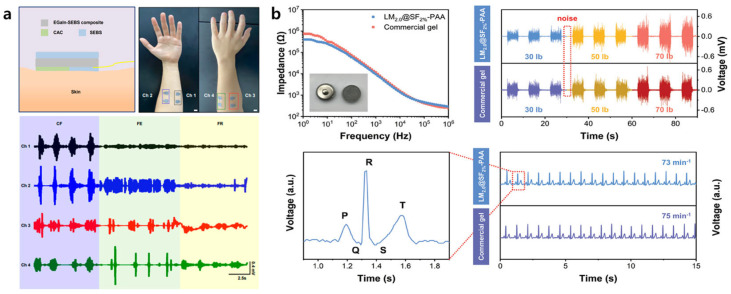
Applications of LM composites in electrophysiological signal monitoring. (**a**) A schematic diagram (upper left) showing the overall structure of an EMG sensor, along with optical images (upper right) showing the multichannel electrode on the human arm, and the EMG signals (bottom) recorded in response to human actions such as clenching the fist (CF), finger extension (FE), and finger relaxation (FR) [70]. (**b**) The comparative interfacial impedance values (upper left), EMG signals (upper right), and ECG signals (bottom) of LM@SF-PAA hydrogel and commercial gel electrodes [71].

Electrophysiological signals such as electrocardiograms (ECGs) and electromyograms (EMGs) provide indirect yet valuable insights into internal physiological activity. These signals play a vital role not only in clinical diagnosis but also in human–machine interfaces (HMIs) [73]. One of the most critical challenges in acquiring high-quality and stable bioelectrical signals is that of achieving strong and consistent adhesion to the skin (Figure 4).

Hydrogels with biocompatibility and ionic conductivity have been extensively investigated as ideal materials for bioelectrical signal acquisition [74]. Given that the adhesive strength of hydrogels is vulnerable to moisture and environmental conditions, hydrogel adhesives play a critical role [75]. Recently, bioadhesives based on biopolymers such as fibrin, gelatin, silk, chitosan, and alginate, which exhibit excellent biocompatibility and biodegradability, have been widely utilized [76]. For example, Kim et al. [70] proposed a wearable EMG sensing system that combines a stretchable EGaIn–styrene–ethylene–butylene–styrene (EGaIn–SEBS) electrode with a chitosan–alginate–chitosan (CAC) hydrogel adhesive layer (Figure 4a). The EGaIn dispersed within the SEBS matrix maintained stable conductivity under strains of up to 520%, while the CAC hydrogel exhibited a high adhesive strength of 15.18 kPa and a low impedance in the low-frequency (1–100 Hz) range, thus resulting in high-quality EMG signal acquisition. Using this setup, the researchers successfully performed multi-channel signal classification of complex muscle movements by placing electrodes on different target muscle groups during various dynamic activities. Meanwhile, Yan et al. [71] developed a high-performance hydrogel using LM@silk fibroin (SF) peptide core–shell particles. The SF coating suppressed oxidation of the LM in aqueous solution and stabilized free radicals, thereby allowing the in situ polymerization of acrylic acid (AA) without requiring additional crosslinkers. The resulting LM@SF–PAA hydrogel demonstrated a lower interfacial impedance along with a higher signal-to-noise ratio (SNR) of 29.8 dB in EMG measurements compared to 23.4 dB for commercial Ag/AgCl electrodes (Figure 4b). Furthermore, a lightweight (~7 g) ECG monitoring patch fabricated using this hydrogel enabled continuous monitoring for 12 h. High signal fidelity was maintained throughout various user movements, including sitting, standing, and walking.

Although various strategies such as double hydrophobic coatings and the incorporation of salts have been developed to improve the water retention capacity of hydrogels for long-term use, their integration with LM composites has not yet been reported and warrants further investigation [77,78].

## 5. Conclusions

LMs, with their unique physicochemical properties, high electrical conductivity, and mechanical flexibility, offer new possibilities in the field of stretchable electronics. Compared to bulk LMs, LMPs provide greater versatility and have emerged as promising fillers for stretchable and conductive composites. When combined with conventional solid fillers, LM-based hybrid fillers can overcome the limitations of single-phase systems to deliver enhanced performance with reduced filler contents while maintaining high stability and reliability. Hybrid LM composites also enable the formation of conductive networks without additional sintering steps. Furthermore, when endowed with responsiveness to external stimuli (e.g., magnetic fields), these composites can provide greater design freedom for device patterning. The development of biodegradable, recyclable, and multifunctional LM composites, particularly those with electromagnetic interference (EMI) shielding capabilities, will be critical for future commercialization in eco-conscious technologies. From an application perspective, LM composites have been successfully implemented in wearable sensing platforms for strain, pressure, electromyogram (EMG), and electrocardiogram (ECG) monitoring. Their inherent advantages, such as high sensitivity, low hysteresis, and outstanding stretchability, make them highly suitable for real-time biological signal monitoring. In particular, hybrid LM/hydrogel systems enable stable, long-term signal acquisition.

Looking forward, LM composites are expected to evolve beyond sensing materials into broader applications such as soft robotics and electronic skin. However, current technologies remain at a relatively early stage of maturity. Continued efforts in materials design and process optimization will be necessary, along with systematic investigations into biocompatibility and long-term stability. As research in this area becomes more diversified and refined, LM-based stretchable conductive composites are anticipated to become key materials in next-generation wearable healthcare devices. 

## Figures and Tables

**Table 2:** The formation of LMPs [13,31,38].

Processing Technique (Droplet Size)		Principle	Advantages	Disadvantages
TOP-DOWN	Drop-on-demand (Tens of µm to a few mm)	Extruding LMs from a syringe or nozzle	3D structure formation	Limited by the minimum size. Precise pressure control required
	Molding (Tens of µm to a few mm)	Pressing into pre-patterned mold	Monodispersity. Large area patterning	Complete filling required. Difficulty in detaching from mold
	Microfluidics (50–200 µm)	Balance between interfacial tension and viscous force	Reliable and repeatable method	Complex system design. Limited by the minimum size
	Sonication (Tens of nm to a few µm)	Fragmentation by sonication	Easy formation of NPs	Polydispersity. Heat generation
	Shearing (A few nm to a few µm)	Application of shear stress	Easy formation of NPs	Polydispersity
BOTTOM-UP	Thermal evaporation (5–150 nm)	Condensation after evaporation of Ga	Size controllability	Ultra-high vacuum required
	Hot injection (12–46 nm)	Growth of Ga precursor	Monodispersity	Low reproducibility

**Table 3:** A summary of LM-based stretchable and conductive wearable composite sensors.

LM Composite	Fabrication Method	Deformation Range	Application	Ref.
LM/PPy/TPU	Electrospinning and polymerization	Up to 135.5% stain	Strain sensing (GF = 4.36 at 0–12.5% strain)	[67]
LM/CNT/PDMS	Blending	Up to 144.33% strain	Strain sensing (GF = 5.35 at 50–100% strain)	[56]
PE/CNT/LM/PE	Laser-assisted coating	Up to 30% compressive strain	Pressure sensing (GF = 57 at 30% strain)	[68]
Porous LM/Ni/PDMS	Blending	Up to 8.9 MPa pressure	Pressure sensing (0.306 kPa^–1^ at 50 kPa)	[69]
LM-SEBS/CAC hydrogel	Drop casting	Up to 520% strain	EMG recording	[70]
LM@SF-PAA hydrogel	Polymerization	Up to 1050% strain	EMG/ECG recording	[71]

## Data Availability

Not applicable.
